# Double Pathology in Rasmussen Encephalitis

**DOI:** 10.15844/pedneurbriefs-34-7

**Published:** 2020-03-07

**Authors:** Iga A. Fudyma, Nitin R. Wadhwani

**Affiliations:** 1 Midwestern University, Downers Grove, IL; 2 Department of Pathology and Laboratory Medicine, Ann & Robert H. Lurie Children’s Hospital of Chicago, Chicago, IL

**Keywords:** Rasmussen Encephalitis, Double Pathology, Epilepsy

## Abstract

Investigators from Children’s Hospital Colorado and University of Colorado conducted a retrospective review of electronic medical records to identify all Rasmussen Encephalitis (RE) cases that had undergone surgery with subsequent pathologic evaluation at Children’s Hospital Colorado during 2005-2019 to determine the frequency of double pathology.

Investigators from Children’s Hospital Colorado and University of Colorado conducted a retrospective review of electronic medical records to identify all Rasmussen Encephalitis (RE) cases that had undergone surgery with subsequent pathologic evaluation at Children’s Hospital Colorado during 2005-2019 to determine the frequency of double pathology. Eleven patients (with a total of 13 resections) were included in the study, 7 of which presented with double pathology.

The most common secondary microscopic abnormality found was focal cortical dysplasia with a predominance of type Ia and type IIa morphology. The investigators documented one case that contained leptomeningeal neuronal heterotopia and another case that contained leptomeningeal melanocytic nevus, both of which have not been previously reported in association with RE. They also noted that areas with dysmorphic neurons or abnormal cortical lamination have more prominent inflammation. The investigators concluded that double pathology may be prevalent in RE, but due to the subjective nature of several of the secondary histologic findings, there may be substantial interobserver variability that imposes limitations on evaluating the frequency of double pathology in RE. [1]

COMMENTARY. The estimated incidence of RE is 2.4 cases per 10 million people under the age of 18. [2] The reported frequency of double pathology in RE is highly variable, as some investigators observed it in less than 10% of their cases [3], while others observed it in 100% of their cases [4]. The literature predominantly consists of isolated case reports and case series from single institutions, and to date, a standardized, large sample, multi-institution study has not been performed to generate a reliable frequency of double pathology. Therefore, it is difficult to draw conclusions about the relationship between cases with and without double pathology in RE.

In non-RE cases, focal cortical dysplasia is diagnosed upon resecting and evaluating a discrete lesion that contains a presumed epileptogenic zone, and it typically does not contain inflammation. In contrast, RE resections tend to be larger (often hemispheric) with diffuse cortical atrophy and inflammation. There is no standardized approach for evaluating secondary pathology occurring in RE since the entire hemisphere is epileptogenic and does not necessarily contain a single, discrete lesion. Some institutions may describe the presence of these secondary microscopic abnormalities, rather than assigning an ILEA classification. When cortical dysplasia is well-defined, one may be more willing to include it as a concomitant diagnosis.

As molecular diagnostics become more accessible and widely utilized, there will be more clarity and congruence in annotating the previously variable reporting of pathology in RE. It may be beneficial to create a registry, biorepository, or a centralized database to provide open access resources to aid in determining the true prevalence of double pathology and pathogenesis of RE. This would be an invaluable asset for researchers as it would provide an opportunity to study a greater number of these rare cases that would otherwise not be available at a single, tertiary institution.

## Disclosures

The authors have declared that no competing interests exist.
